# Effects of the Visual Exercise Environments on Cognitive Directed Attention, Energy Expenditure and Perceived Exertion

**DOI:** 10.3390/ijerph120707321

**Published:** 2015-06-30

**Authors:** Mike Rogerson, Jo Barton

**Affiliations:** Centre for Sports and Exercise Science, School of Biological Sciences, University of Essex, Wivenhoe Park, Colchester, Essex, CO4 3SQ, UK

**Keywords:** green exercise, cognitive functioning, wellbeing, directed attention, exercise environments, perceived exertion

## Abstract

Green exercise research often reports psychological health outcomes without rigorously controlling exercise. This study examines effects of visual exercise environments on directed attention, perceived exertion and time to exhaustion, whilst measuring and controlling the exercise component. Participants completed three experimental conditions in a randomized counterbalanced order. Conditions varied by video content viewed (nature; built; control) during two consistently-ordered exercise bouts (Exercise 1: 60% VO2peakInt for 15-mins; Exercise 2: 85% VO2peakInt to voluntary exhaustion). In each condition, participants completed modified Backwards Digit Span tests (a measure of directed attention) pre- and post-Exercise 1. Energy expenditure, respiratory exchange ratio and perceived exertion were measured during both exercise bouts. Time to exhaustion in Exercise 2 was also recorded. There was a significant time by condition interaction for Backwards Digit Span scores (F_2,22_ = 6.267, *p* = 0.007). Scores significantly improved in the nature condition (*p* < 0.001) but did not in the built or control conditions. There were no significant differences between conditions for either perceived exertion or physiological measures during either Exercise 1 or Exercise 2, or for time to exhaustion in Exercise 2. This was the first study to demonstrate effects of controlled exercise conducted in different visual environments on post-exercise directed attention. Via psychological mechanisms alone, visual nature facilitates attention restoration during moderate-intensity exercise.

## 1. Introduction

Green exercise is defined as engaging in ‘physical activities whilst being directly exposed to nature’ [1]. Green exercise research has adopted three different approaches for comparing both physiological and psychological exercise outcomes. Some research compares outcomes of built- *vs.* nature-based outdoor exercise [2,3,4,5,6]. Other research compares outcomes of indoor exercise to those of outdoor exercise [7,8,9]. A third approach uses ergometers within laboratory settings to control the exercise performed and examine the importance of the visual exercise-environment [1,10,11]. Collectively, compared to non-green exercise, green exercise improves psychological measures such as mood, self-esteem and vitality [7,8,9,10,11,12], and physiological markers such as blood pressure and heart rate variability (HRV) [1,2,3,4]. However, some conflicting findings suggest that influences of exercise-environments on psychological outcomes may be overstated [13], and findings in adult samples have not been mirrored in adolescent samples [11]. Additionally, often convenience methodologies used do not decipher relative contributions of the respective exercise and environment components [14,15].

“Directed attention” is another psychological outcome that can be influenced by exercise-environment selection [5,6]. Directed attention is the effortful cognitive ability to avoid being distracted by competing stimuli [16,17]. Brain regions associated with processes of mental effort, attention and mediation of cognitive control can fatigue over time [16]. This depletion of directed attention is termed directed attention fatigue. Using involuntary attention—that which does not involve mental effort [5,18], decreases use of directed attention. This provides opportunity for restoration of depleted attentional resources [16].

Transient hypofrontality [19,20,21] posits a pathway linking exercise with attention restoration. Prefrontal cortex activation is associated with processes of directed attention [22,23,24,25]. Exercise facilitates prefrontal cortex restoration via transient hypofrontality [19,20,21], that is, decreases in prefrontal cortex activity that occur in conjunction with increased motor cortex activity. *During* exercise, this decreased prefrontal cortex activity may be detrimental to cognitive performance [20,26]. However, *following* prolonged opportunity for prefrontal cortex restoration, transient hypofrontality improves post-exercise executive functioning and cognitive performance [27,28]. 

Disparate to exercise-related mechanisms, attention restoration theory proposes that visual characteristics of natural environments promote involuntary attention and facilitate restoration of directed attention capacity and affective states [18,29,30]. Spending an hour at rest in an outdoor garden facilitates directed attention improvements in elderly individuals, compared to equivalent rest in a favorite indoor room [31]. Viewing photographs of nature environments improves directed attention-related task performance, whereas viewing urban environments does not [32]. Within workplace settings, “micro-restorative experiences” provided by views of nature through a window or the presence of plants indoors can reduce directed attention fatigue [33,34].

Green exercise promotes a greater psychological engagement with nature than does viewing nature [35]. Greater immersion may elicit a fuller experience and greater responses to nature environments [36]. Such a role of immersion suggests that green exercise might provide greater scope for attention restoration than either micro-restorative experiences or time spent at rest in nature environments. Additionally, the disparate influences of exercise and of nature environments may positively interact in a manner that further promotes attention restoration. Directed attention improves following a walk in nature environments but not after an equivalent walk around more built routes [5,6]. However, the variable of social presence of others was not controlled in this research. Additionally, although Hartig *et al.* [6] attempted to control exercise intensity via researchers leading participants in order to maintain a slow walking pace with stops at specified locations on route, exercise intensity (defined as the total or rate of energy expenditure during exercise [37,38]) was not rigorously controlled in these studies. This is important because intensity influences psychological outcomes of exercise including cognitive performance [39,40,41,42].

Exercise environment also influences perceived exertion (how hard one feels that they are physically working during activity; measured by Rated Perceived Exertion scale [43,44,45]). This is one possible pathway via which a lack of control of the exercise component can promote problematic differences in exercise intensity between experimental conditions. When exercising at an instructed perceived exertion, individuals work harder (measured by speed, heart rate and blood lactate concentration) during outdoor exercise than during indoor treadmill exercise [46]. Concurrently, during self-paced exercise individuals walk faster and work harder (measured by heart rate) yet report lower perceived exertion during outdoor walking compared to indoor treadmill walking [7]. These findings are concurrent with the notion that synthetic environments demand greater directed attention processing [18,29,30,47], as cognitive fatigue promotes greater perceived exertion and impairs performance of exhaustive exercise [48]. However, biomechanical and climatic differences between indoor and outdoor exercise are not controlled within these research designs, therefore origins of reported effects are unclear [7,46]. The color of visual environment is important to perceived exertion during exercise. Individuals report significantly lower perceived exertion during cycling exercise whilst viewing a nature-scene video, compared to cycling whilst viewing either achromatic or red-filter versions of the same video [10]. However, it is not known how perceived exertion varies with different visual exercise-environment *types* during controlled exercise. 

Lacking control of the exercise component in these studies is also problematic for understanding the origins of reported influences on *physiological* outcomes. It is reported that walking in nature environments promotes lower post-exercise heart rate, greater HRV, lower blood pressure and lower sympathetic nerve activity than equivalent walks in built environments [4,49,50]. Environment-associated differences in physiology as measured during exposure have also been reported [4]. However, uncontrolled exercise intensity means that findings from these studies cannot be confidently attributed to environment alone. Physiological effects of environment are yet to be demonstrated during controlled exercise. Heart rate, VO_2_ and respiratory exchange ratio are not significantly influenced by color properties of visual exercise environment during controlled cycling exercise [10]. However, influences of holistic visual exercise-environments on directed attention have not been examined during controlled exercise. 

The design of the current study enables examination of the reported influence of exercise-environment on perceived exertion [7,10,46]. Biological links between subjective sensations of effort and exercise-related physiology comprise a mechanism by which perceived exertion contributes to limiting of exercise performance [51,52]. Perceived exertion is strongly related to individuals’ time to exhaustion during high-intensity exercise [53]. The current study examines whether visual exercise-environment influences perceived exertion and time to voluntary exhaustion during high-intensity exercise.

The current study measured pre- to post-exercise changes in directed attention in three conditions: a nature condition, a built condition and a control condition. Energy expenditure, respiratory exchange ratio and heart rate were measured to ensure: (i) comparability of the exercise component between conditions; (ii) identification of physiological effects of visual environments *during* exercise, and (iii) impacts of physiological differences on changes in cognitive function. The primary hypothesis is that (i) directed attention will improve more after a 15-mins exercise bout whilst viewing video footage of a nature environment, compared to footage of a built environment or viewing a blank screen (control). Secondary hypotheses include (ii) during a steady-state 15-mins exercise bout there will be no significant differences in participants’ energy expenditure, respiratory exchange ratio and heart rate between conditions; (iii) perceived exertion will be lower and time to exhaustion will be longer in the nature condition than in a built condition or control condition.

## 2. Methods 

### 2.1. Participants

Twelve healthy adult participants (6 male, 6 female; age 27.8 ± 5.5 years; stature 173.4 ± 11.9 cm; mass 65.4 ± 10.5 kg) were recruited via oral and written advertisements, from student and staff populations at the University of Essex. Participants were instructed to avoid alcohol, caffeine and strenuous exercise within 24-hours prior to each test occasion, and to maintain usual lifestyle and exercise routines throughout their participation. Participants provided written informed consent. Ethical approval for this study was obtained from the School Ethics Committee.

### 2.2. Design 

A within-subjects design was used. Participants completed a Bruce treadmill protocol [54,55] on the first of four test occasions. Participants then completed three experimental test occasions in a randomized, counter-balanced order. Time of day was consistent between occasions (within 2-hours) and occasions were a minimum of 7-days apart, to avoid fatigue effects. Average time between conditions was 13-days. Maximum time between conditions was 25-days.

Experimental test occasions differed only by the video-footage displayed on a screen during exercise. In the nature condition, the video consisted of scenes extracted from “Evening Run through Endless Forest”. In the built condition, the video consisted of scenes extracted from the “Boston Marathon Route” (this footage follows the marathon route but is not filmed in the context of the Boston marathon; both videos produced by Outside Interactive, Hopkinton, MA, USA). The speed of movement was consistent (9.62 km/h) between videos, to avoid confounding variations in pacing-deception and optical flow. To ensure that participants’ semantic associations with the city of Boston or the Boston marathon were not a confounding influence; participants were asked whether they recognized the place in the footage. No participants recognized the place. In order to reduce potential social influences, footage was edited to minimize presence of others. For the video footage of the built condition, scenes from the “Boston Marathon Route” video during which the route moves through areas outside of the city center, which are of greater greenery, were not included in the footage used. However, some elements of greenery were present in the footage used; for example, trees lining pavements in the city area. The footage used both provided contrast between built and nature conditions and avoided potential confounding influences of large quantities of greenery in both the built condition and the nature condition. The presence of some but little greenery is typical of modern urbanized city areas, thus maintaining the ecological validity of the current study. In the control condition, no video footage was displayed on the screen. That is, participants viewed a blank white screen. The video footage did not include auditory sound, so to isolate the independent variable of visual environmental stimuli.

### 2.3. Procedures 

#### 2.3.1. First Test Occasion

Participants completed Physical Activity Readiness Questionnaire and Informed Consent documents and were briefed on the session’s content. Resting blood pressure was then measured in the seated position (using an automatic blood pressure unit; MX3 Plus; Omron, Illinois, USA). Participants were then familiarized and fitted with a heart rate monitor (FR-70 Garmin, Olathe, USA) and portable gas analyzer (K4b^2^: Cosmed, Rome, Italy).

Participants completed a 5-min warm-up which consisted of a 2:30-mins treadmill walk (JW200 Power jog, Sport Engineering Ltd, Birmingham, UK) at a self-selected speed to reflect 50% of perceived maximum effort; followed by a set of stretching exercises as directed by computer presentation (participants were instructed to stretch to an extent that they felt to be at “8 out of 10” in relation to their maximum).

Participants then performed a Bruce treadmill protocol to exhaustion. This was to identify an intensity (speed and gradient) that elicited participants’ VO_2_peak (VO_2_peakInt), for application in the experimental protocols. The treadmill’s display screen was covered throughout the study, in order to prevent subjects from viewing intensity, distance and time information.

#### 2.3.2. Experimental Conditions

Participants’ blood pressure was measured; they were briefed regarding the session’s content, and fitted with heart rate and portable gas analysis equipment. Next, participants performed a set 5-min warm-up consisting of a 2:30-mins treadmill exercise—at 50% VO_2_peakInt, followed by the same set of stretching routine as in the first test occasion. 

Participants sat at a desk and completed a directed attention-reducing battery in order to utilise and thereby reduce their remaining directed attentional capacity, followed by a Backwards Digit Span test. During both the directed attention-reducing battery and the Backwards Digit Span test, the mask of the portable gas analyser was worn loosely around the neck.

Participants then completed a 15-min bout of exercise on the treadmill, at 60% VO_2_peakInt (Exercise 1). Participants were instructed to engage with the content of the videos during the exercise. Perceived exertion was measured at 4:30-, 9:30-, and 14:30-mins. 

Participants then completed a second Backwards Digit Span test. The warm-up was completed before the directed attention-reducing battery in order to enable the pre- and post-Exercise 1 Backwards Digit Span tests to occur directly before and after the main manipulation of the study—viewing different environmental scenes during a 15-min exercise bout. This was to ensure that differences between conditions in participants’ lived experiences of the warm-up did not influence performance on post-Exercise 1 Backwards Digit Span tests. 

Participants then returned to the treadmill to complete a second exercise bout (Exercise 2). Intensity of Exercise 2 was 85% VO_2_peakInt and participants were instructed to run to voluntary exhaustion. Perceived exertion was measured at 2-min intervals. Time to exhaustion for Exercise 2 was also recorded. As variation in participants’ time to exhaustion may have served as a confounding influence between conditions, no Backwards Digit Span tests were completed following Exercise 2.

### 2.4. Measures 

#### 2.4.1. Directed Attention-Reducing Battery 

Two tasks (Subtraction of Serial Sevens Test and spelling words backwards) modified from the Mini Mental State Examination [56] were completed to utilize and deplete participants’ directed attention. Multiple variations were used in order to comprise a 5-min directed attention-reducing battery. For Subtraction of Serial Sevens Test, participants were asked to verbally subtract seven from a randomly generated number between 100 and 999. They continued verbally counting down until reaching six or less before restarting with another number, for 2:30-mins. For spelling words backwards, participants were instructed to listen to the experimenter read and then spell (forwards) an 8-letter word, before attempting to verbally spell that word in reverse. Each word was used only once, to avoid learning effects. This process continued for 2:30-mins.

#### 2.4.2. Backwards Digit Span Test (Modified)

The Backwards Digit Span test [57] requires attentional effort to mentally hold, track and rearrange items within the short-term memory [5,58]. It has frequently been used as a measure of directed attention [5,16,59,60,61].

Backwards Digit Span testing often requires participants to listen to strings of numbers before attempting to verbally recite them in reverse order [5]. In the current study, participants viewed number strings displayed on a computer screen before writing their answers by hand when prompted; ensuring consistency with the experiment’s focus on the sense of vision. For each string, numbers were presented serially on the screen, for one second each. Rules were instructed to the participants both on the screen and verbally by the experimenter, at the start of each test. These were: do not write anything down until prompted to by the screen; when you are writing down your answer, you must physically write in the direction of from left to right; do not mouth the numbers at all, at any time; if you cannot remember, please make the best guess that you can. The experimenter was positioned in order to view the screen, the participant and the answer sheet, to ensure that participants adhered to the given rules.

In a standard Backwards Digit Span test, the length of the number-string increases by 1 and continues until participants fail two consecutive attempts at reciting strings of a given length—generating a score in relation to the maximum string-length successfully recited [57]. Participants attempted to recite nine number-strings which were 3–11 digits in length, increasing with order, via a programmed computer-based presentation. The set duration of each Backwards Digit Span test ensured consistency of time between the Exercise 1 and Exercise 2, between participants and conditions. 

#### 2.4.3. Respiratory Exchange Ratio 

Respiratory exchange ratio is calculated as = VCO_2_/VO_2_ and gives an indication of the energy source being utilized by the working tissues of the body. Respiratory exchange ratio values above 1.1 can be used as an endpoint criterion during VO_2_max and VO_2_peak tests [62].

### 2.5. Data Treatment 

The shortest recorded time to exhaustion for Exercise 2 was 4:47-mins. Therefore, to ensure comparability of data both between participants and between conditions, for Exercise 2, only the initial 4:47-mins of physiology data (energy expenditure, respiratory exchange ratio, and heart rate) were analyzed. Heart rate data for two participants was excluded from analysis due to equipment failure.

### 2.6. Statistical Analysis 

IBM SPSS Statistics for Windows, version 19 [63] was used for statistical analyses. One-way repeated-measures ANOVAs were performed to check for differences in: (i) pre-exercise Backwards Digit Span test scores between conditions; (ii) mean heart rate values between conditions during Exercise 1 and Exercise 2; (iii) perceived exertion at each of three time points (4:30-mins, 9:30-mins, 14:30-mins); and (iv) time to exhaustion during Exercise 2. A two-way repeated measures ANOVA was performed on Backwards Digit Span test scores in order to identify any time-by-condition interaction effect. As energy expenditure and respiratory exchange ratio are directly related variables, one-way repeated measures MANOVAs were used to analyze these variables for each of Exercise 1 and Exercise 2. Although heart rate is also a related physiological variable, this was analyzed separately in order to maintain a sample of 12 for the variables of energy expenditure and respiratory exchange ratio. An alpha level of 0.05 was used to indicate statistical significance.

## 3. Results

### 3.1. Backwards Digit Span Test Scores

**Figure 1:**
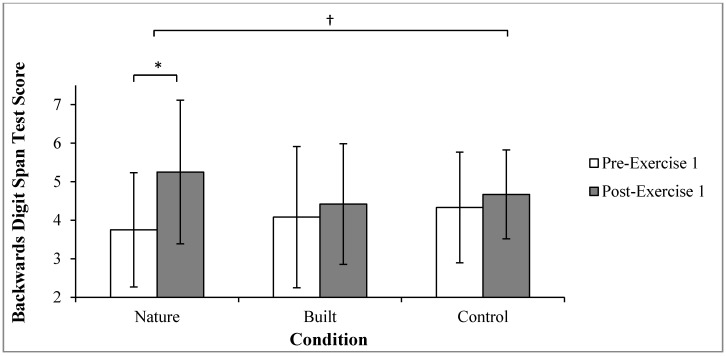
Mean (±SD) pre- and post- Exercise 1 Backwards Digit Span test scores by condition; Higher score represents greater level of directed attention; *, pre- and post- Exercise 1 values significantly differ (*p* = 0.001); **^†^**, significant time by condition interaction (*p* = 0.007).

Results of a one-way repeated measures ANOVA indicated no significant effect for condition on pre-exercise Backwards Digit Span test scores (*p* = 0.290). A two-way repeated measures ANOVA indicated a significant interaction effect for time by condition (F_2,22_ = 6.267, *p* = 0.007, ηp^2^ = 0.363; Figure 1). Pairwise comparisons showed Backwards Digit Span improvements were statistically significant in nature condition (*p* < 0.001, 95% CI (0.87, 2.14)), but were not in the built condition (*p* = 0.266) or control condition (*p* = 0.166).

### 3.2. Physiological Measures

**Figure 2:**
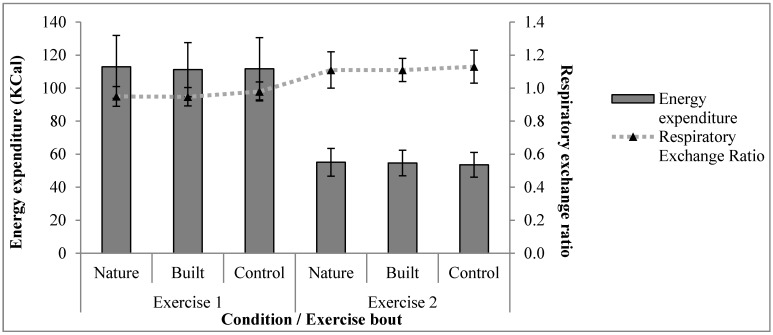
Mean (±SD) energy expenditure and respiratory exchange ratio by condition for Exercise 1 and Exercise 2; Exercise 1 duration was 15-mins, Exercise 2 values each represent 4:47-mins of data.

One-way repeated measures MANOVAs indicated that there was no significant effect of condition on energy expenditure and respiratory exchange ratio for either Exercise 1 (*p* = 0.34) or Exercise 2 (*p* = 0.63). Energy expenditure and respiratory exchange ratio values are shown in Figure 2. One-way repeated measured ANOVAs indicated no statistically significant effect of condition on mean heart rate values in either Exercise 1 (*p* = 0.36) or Exercise 2 (*p* = 0.88; Table 1). 

**Table 1:** Mean (± SD) results for heart rate and time to exhaustion by condition.

Measure	Exercise Bout / Time (mins)	Condition		
		Nature	Built	Control
Heart Rate (bpm)	Exercise 1	108.8 ± 9.2	109.9 ± 11.2	107.5 ± 12.0
	Exercise 2	145.7 ± 7.9	145.1 ± 11.0	144.1 ± 9.5
Time to exhaustion (secs)	Exercise 2	824.1 ± 336.3	726.0 ± 269.8	769.17 ± 292.2

### 3.3. Perceived exertion and Time to Exhaustion 

**Figure 3:**
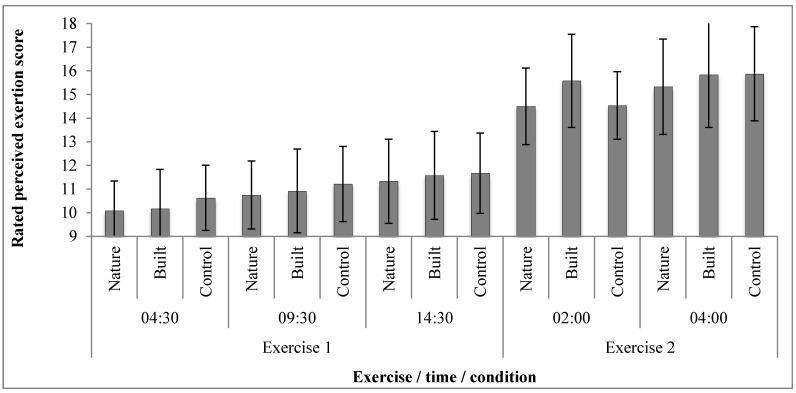
Perceived exertion scores by exercise bout, time and condition; Rated perceived exertion: minimum value = 6 (no exertion at all), maximum value = 20 (maximal exertion).

One-way repeated measures ANOVAs indicated that there were no significant differences in perceived exertion scores between conditions during Exercise 1 at either 4:30-mins (p= 0.389), 9:30-mins (*p* = 0.509) or 14:30-mins (*p* = 0.655), or during Exercise 2 at either 2-mins (*p* = 0.947) or 4-mins (*p* = 0.302). Perceived exertion values are presented in Figure 3. Although time to exhaustion was longest in the nature condition, results of a one-way repeated-measures ANOVA indicated no statistically significant main effect for condition (*p* = 0.203; Table 1).

## 4. Discussion

The purpose of this study was to examine effects of visual exercise-environments on directed attention whilst controlling the exercise component. Secondary aims were to investigate reported effects of visual exercise-environments on measures of energy expenditure, respiratory exchange ratio, heart rate, perceived exertion and time to exhaustion. 

### 4.1. Directed Attention

Concurrent with previous findings relating to transient hypofrontality, directed attention improved from pre- to post-exercise in all conditions [19,20,21]. However, it is not possible within the current study to confirm this mechanism. Without inclusion of a non-exercise control condition, these acute improvements might also be considered to be short-term learning or motivational effects. The primary hypothesis that directed attention would improve more after a 15-min exercise bout whilst viewing video footage of a nature environment, compared to footage of a built environment or viewing a blank screen (control), was supported. Significant improvements in directed attention were observed in the nature condition but did not in the built or control conditions. This finding is concurrent with that of Berman *et al.* [5]. One possible explanation for this finding is that the nature video reduced the need for directed attentional processes, maximizing attention restoration during the opportunity afforded by Exercise 1 [16,18,30]. To the authors’ knowledge, this is the first study to demonstrate an effect of visual environment on directed attention whilst controlling exercise. This is also the first study to combine measures of attention following steady-state exercise with measures of perceived exertion and time to exhaustion during vigorous exercise. A strength of this study was that it rigorously controlled exercise between conditions. Exercise intensities (speed/gradient) of Exercise 1 and Exercise 2 were replicated via programming of a treadmill, to ensure that the independent variable of visual environment was isolated. Unlike previous research [5,6,59], it is possible to conclude that visual nature facilitates attention restoration during exercise independently from influences of exercise.

Attention restoration theory postulates that restoration in turn promotes improvements in affective states [18]. The results of the current study support the possibility that attention restoration might partly underpin the influence of exercise-environments on affective states [15,64,65]. Future research should investigate this possibility further via paradigms that include pre- and post-exercise measures both of directed attention and affect whilst controlling or measuring the exercise component. 

### 4.2. Physiological Measures 

All participants’ respiratory exchange ratio values for the final minute of the Bruce protocol were above 1.1, indicating that identified VO_2_peaks likely represented maximal effort [62]. Greater respiratory exchange ratio values for Exercise 2 than for Exercise 1 are a product of the exercise intensities performed by participants during the respective exercise bouts.

The secondary hypothesis that during a steady-state 15-min exercise bout there would be no significant differences in participants’ energy expenditure, respiratory exchange ratio and heart rate between conditions was supported. Previously reported physiological differences during exercise in different environments may have been due to differences in exercise [4]. Considered together with previously reported effects of exercise-environments on post-exercise physiology, the results suggest that physiological effects of exercise-environments may occur primarily during recovery from exercise [4,49,50,66]. If physiological effects of exercise-environments occur during exercise, this result alludes to two further possibilities: (i) that physiological responses to visual environments were *overridden* by exercise-related physiology; (ii) that physiological responses to visual environments were *masked* by exercise-related physiology. A limitation of the current study is that its results do not indicate whether either of these possibilities is true, or occurred. 

The current study’s design controlled individual-, exercise- and environment-related variables except for visual stimuli. Psychological effects of visual exercise-environments reported by both the current study and previous laboratory-based research likely resulted via psychological mechanisms alone [1,10]. Mechanisms of this kind, such as attention restoration and stress reduction have been theorised [16,18,29,47]. Furthering understanding of perceptual and cognitive interactions with environments during exercise can support maximization of mental and physical health outcomes from exercise participation, such as lowering blood pressure [1,2] and improving mood [14] and perceived mental health [2].

### 4.3. Perceived Exertion 

The results did not support the secondary hypothesis that perceived exertion would be lower and time to exhaustion would be longer in the nature condition than in a built condition or control condition. The current study failed to support previously reported findings that exercise-environments influence perceived exertion, although it is notable that different exercise modes (walking, cycling) were used in these previous studies [7,10]. Large standard deviations indicate that statistical under-powering of this study was the likely reason that despite time to exhaustion in nature condition being 12.7% greater than in the built condition and 6.9% greater than in the control condition, differences were not statistically significant. Indeed a major limitation of the current study was its small sample size, which may have served to increase type I and type II statistical errors in the analyses of each of the reported dependent variables. The results indicate that future research should investigate these variables using designs that are sufficiently powered following sample-size calculations.

Visual exercise-environment influenced one psychological measure of this study (Backwards Digit Span score) but not the other (perceived exertion). To speculate here, one possible explanation of this might be related to the face mask worn during the exercise (as part of the portable gas analyser). Amplified sounds of breathing may have facilitated internal focus on physiological sensations, aiding consistency in perceived exertion [67,68]. This may have reduced opportunity for visual exercise-environment to influence participants’ appraisal and reporting of exertion. Although mask-related cognitions may have also functioned to reduce opportunity for environment-related cognitions, as many aspects of restoration-related processes occur subconsciously [18], attention restoration via visual exercise-environment may have therefore remained unhindered. 

In order to examine influences of visual environmental stimuli, no auditory stimuli were included in the video footage of the nature and built conditions. This may have caused confounding dissonance within each of the nature and built conditions in that such visual stimuli would normally be accompanied by appropriate auditory stimuli. It is not known in which condition dissonance may have been greatest, however, it seems likely that the control condition would elicit least dissonance due to its lack of visual cues. Future work might examine the importance of different sensory modalities to influences of exercise-environments of this kind. Additionally here, whereas nature and built conditions included video content, the control condition did not include such visual cues. Providing neutral stimuli for participants to visually attend to would have avoided this potential confound. However, the control condition served as a comparison to enable influences of treadmill exercise alone to be examined.

### 4.4. Application 

The findings further previous research which has examined the notion that green exercise might promote outcomes beneficial to workplace contexts [2]. Acute bouts of green exercise promote restoration from directed attention fatigue, thus replenishing an important resource for performance of cognitively demanding workplace tasks. The current findings are also relevant to educational contexts. Attention restoration via green exercise environments can aid children’s concentration in school and ameliorate symptoms of ADHD [59,69]. Results of the current study suggest that the visual element of real green exercise environments may play an important role in obtaining these benefits.

## 5. Conclusions 

This was the first study to demonstrate effects of controlled exercise conducted in different visual environments on post-exercise directed attention. Visually perceived nature promotes attention restoration during moderate intensity exercise, independent of physiological influence. Additionally to physiological and other psychological outcomes [1,3,4,12,15,64,65,66,70], acute green exercise participation benefits cognitive functioning [5,6]. Such improvements are relevant for workplace and educational performance. Equipment-related cognitions may have limited potential influences of visual exercise-environments on perceived exertion, and in turn, time to exhaustion.
